# Optimizing the role of ambulatory chemotherapy in response to the Covid-19 pandemic

**DOI:** 10.1177/1078155220963527

**Published:** 2020-10-07

**Authors:** Racha Sabbagh Dit Hawasli, Shereen Nabhani-Gebara

**Affiliations:** Faculty of Engineering and Computing, School of Pharmacy and Chemistry, 4264 Kingston University, London, UK

**Keywords:** Covid-19, Coronavirus, Ambulatory Chemotherapy, cancer care

## Covid-19 pandemic

On July 4th 2020, the World Health Organization (WHO) issued its 166th Situation Report announcing nearly 11 million confirmed coronavirus disease (Covid-19) cases worldwide, hitting a death toll of nearly over 500,000.^1^ Hospitals are investing immense resources to treat infected patients; many elective surgeries have been halted and strict infection control measures are now in place restricting access to clinics and hospitals.

This pandemic outbreak is not expected to cease anytime soon, and has even engendered shortages in main resources across healthcare systems worldwide.^2,3^

More so, ethical considerations have risen following a retrospective study in China revealing poor survival rates of Covid-19 infected patients with health complications. It is speculated that allocation of health resources will soon be diverted to patients with higher chances of survival to cohere with “greater social good”.^3,4^

## Cancer and Covid-19

This restructuring has hence threatened care provision to patients with serious chronic diseases, such as cancer. Noticeably, cancer patients are in an intricate situation; delayed surgeries and treatments expose them to metastasis and disease progression, while the customary treatments with inherent immunosuppressant properties delivered in-hospital put them further at risk. In this regard, it was noted that 20% of the fatalities from Covid-19 in Italy occurred in patients with an active cancer.^5^ Additionally, reports from China highlighted that infected cancer patients are more prone to require ventilators, ICU admissions or dying compared to others.^6^

## Guidelines on cancer care during the pandemic

Oncologists are facing a thorny risk-benefit situation which pushed oncology societies and authorities to issue guidelines on cancer care during the pandemic.^2,3,7^

The European Society for Medical Oncology (ESMO) advised using telemedicine to reduce clinic visits of cancer patients, as well as shifting to oral and subcutaneous chemotherapy at home when possible.^2,8^

Richard Schilsky, the chief medical officer of the American Society of Clinical Oncology (ASCO), pointed that patients with blood cancer are more vulnerable that those with solid tumours. The observed immunosuppression in the latter group is mainly related to treatment, and thus could be corrected with colony-stimulating factors, while the former ones suffer from an additional direct effect of the disease itself.^2^ A recent publication by Tang et al. found a great correlation between chemotherapy infusions delivered to Covid-19 infected cancer patients and death. Subsequently, ASCO issued a statement calling for cautious risk to benefit assessment on whether to halt or proceed with such a treatment.^9^

NHS England assigned a set of priority groups for cancer patients who are candidates for surgery, systemic chemotherapy and radiotherapy based on high expected treatment outcomes.^2^ This was in correlation with treatment decisions suggested by Ueda et al. encouraging treatments for patients with solid tumours, as well as for those in metastatic states to avoid “loss of the window to treat”. As such, providing treatment on time could prevent overwhelming wards from complications related to disease progression.^3^

The Centers for Disease Control and Prevention (CDC) recommended assessing the suitability of cancer patients’ homes for care provision. This entailed the presence of carers for assistance and the availability of designated patient bedroom, essentials, and personal protective equipment. Finally, it is essential to comply to infection control at home.^7^

## Pragmatic approaches for cancer care continuum

In order to mitigate the impact of Covid-19 on the treatment of cancer patients, a change in practice may ensure greater continuity of care in the setting of a global pandemic.

Ambulatory chemotherapy (AC), entails delivering chemotherapy to cancer patients using lightweight portable infusions pumps outside the hospital. In recent years, the choice of the pump was principally for a disposable elastomeric or battery-operated one. AC is distinct from home chemotherapy which entails delivery of chemotherapy entirely at patients’ homes with the partial support of healthcare professionals.

Patients are assessed for their suitability to receive AC depending on their performance status, the chemotherapy regimen prescribed, the presence of a carer at home, their proximity to a hospital, and sometimes a prior successful infusion at the hospital.

AC was successful in decreasing cost of care, enhancing the quality of life of cancer patients, and empowering them to regain control and normalcy.^10–12^

Additionally, adopting AC would secure hospital beds for patients in need of hospitalization, while treating vulnerable ones from the comfort of their homes, with potentially lower chances of contracting nosocomial infections.^10^

Finally, in a study conducted at a Korean hospital, patients who chose to receive AC scored higher satisfaction levels than those who chose treatment at the oncology ward.^11^ As such, it is important to involve the patients in the decision-making process when planning to adopt AC in order to fully experience its benefits. Furthermore, adequate counselling is essential for patients and their carers in order to deal with any emergency while at home.

Although AC is the standard of oncology care in many high and upper-middle income countries, it is still far from being integrated in many healthcare systems. Nevertheless, during the Covid-19 pandemic, AC is an attractive alternative for cancer patients at times where treatment delays and hospital visits are both critical.

An appealing scheme would be to couple technology enabled care, diagnostics, and AC. Telehealth is now the primary source of consultations in England to restrict patients’ exposure to hospital settings.^2^ Similarly, in its recent comment letter in June 2020, ASCO reinforced the use of telehealth during and beyond the Covid-19 pandemic.^9^

As such, patients would be followed up from the convenience of their homes through telehealth, supported with home testing kits (i.e. point of care devices) to check for markers such as white blood cells counts (WBC), Haemoglobin (Hb), C-reactive protein (CRP), platelets and other necessary tests. It is essential to design a pathway that secures these kits to the patients, then retrieves and delivers the results to the relevant healthcare professionals. These kits are usually made available through the patients’ designated organisation; the Trusts in the UK, or private hospitals in the States which then apply for reimbursement as part of the bundle of care. These kits have a major impact in the care continuum during the Covid-19 era distributed through the mobile care unit (MCU).

The proposed pathway is to have the patients’ files reviewed by the medical team, and if appropriate, a communication would be sent to prepare the MCU for delivery of the treatment. At the time of the appointment, the MCU staff dispenses the treatment and the home testing kits to the designated patients along with appropriate counselling. The MCU is able to deliver oral medications, short intravenous infusions disconnected before patient discharge, or portable infusion pumps for prolonged infusions.

It is important to note that, once this system is established, it could serve other therapeutic areas or drugs. Prolonged infusions of antibiotics are already under study and have shown promising results.^13,14^

There are, however, some key challenges to address for this scheme to be successfully implemented.

First, stability studies are needed to allow extended infusions of chemotherapy drugs at home while retaining their physico-chemical properties. 5-FU, Ifosfamide and Mesna, and Trabectidin are a few examples of drugs with renowned stability data.^15^

However, there remains numerous drugs with unknown stability data and conditions especially with the introduction of the monoclonal antibodies. Thus, to date, a wide range of anti-cancer therapies are not suitable for AC delivery.

More so, it would be wise to visit the recently published update on the “USP General Chapter <797> Pharmaceutical Compounding Sterile Preparations” while preparing pumps for AC administration. This update highlights important considerations for beyond-use dates at times of Covid-19 taking into consideration its impact on the global supply chain of medications and medical supplies such as personal protective equipment (PPE).^16^

Second, point of care devices are essential to allow for testing before treatment initiation; availability, affordability, and patient training are three important pillars for their use.

Last but not least, healthcare reforms are needed to establish this new care pathway as well as train the staff to undertake their new roles. The feasibility for establishing a robust telehealth system to connect patients to their care providers must be well studied.

## Conclusion

The pandemic outbreak of the coronavirus has disrupted the continuum of care for cancer patients; hospitals are under increased pressure to contain the virus at the expense of many services including chemotherapy delivery. Hospital beds, healthcare practitioners and budgets are diverted to contain further escalation of the crisis. Additionally, as high-risk individuals, cancer patients are advised to avoid hospital visits when permissible during such times. Dealing with this unprecedented pandemic requires drastic deviation from previous healthcare delivery schemes. In this perspective, ambulatory chemotherapy and telehealth present as an appealing alternative to hospital visits especially following the endorsement of home treatment under similar conditions. Efforts must be made to support this shift in care and fund research to bridge the current gap. It is wise to apply this scheme to other therapeutic areas as all critical patients are in need of care continuum.
